# A Task Complexity Analysis Method to Study the Emergency Situation under Automated Metro System

**DOI:** 10.3390/ijerph20032314

**Published:** 2023-01-28

**Authors:** Ke Niu, Wenbo Liu, Jia Zhang, Mengxuan Liang, Huimin Li, Yaqiong Zhang, Yihang Du

**Affiliations:** 1 Collaborative Innovation Center for HSR Driver Health and Safety, Zhengzhou Railway Vocational &Technical College, Zhengzhou 451460, China; 2 Henan Engineering Research Center of Rail Transit Intelligent Security, Zhengzhou Railway Vocational &Technical College, Zhengzhou 451460, China; 3 Department of Construction Engineering and Management, North China University of Water Resources and Electric Power, Zhengzhou 450046, China; 4 New Media Arts Department, National Academy of Chinese Theatre Arts, Beijing 100073, China

**Keywords:** emergency situation, automatic metro system, comprehensive method, task complexity

## Abstract

System upgrades and team members interactions lead to changes in task structure. Therefore, in order to handle emergencies efficiently and safely, a comprehensive method of the traffic dispatching team task complexity (TDTTC) is proposed based on team cognitive work analysis (Team-CWA) and network feature analysis. The method comes from the perspective of the socio-technical system. Two stages were included in this method. In the first stage, four phases of Team-CWA, i.e., team work domain analysis, team control task analysis, team strategies analysis, and team worker competencies analysis, were applied in the qualitative analysis of TDTTC. Then in the second stage, a mapping process was established based on events and information cues. After the team task network was established, the characteristic indexes of node degree/average degree, average shortest path length, agglomeration coefficient, and overall network performance for TDTTC were extracted to analyze TDTTC quantitatively. The cases of tasks for screen door fault under grade of automation GOA1–GOA4 were compared. The results revealed that the more nodes and communication between nodes, the larger the network scale was, which would lead to the TDTTC being more complicated no matter what level of automation system it was under. This method is not only the exploration of cognitive engineering theory in the field of task complexity, but also the innovation of team task complexity in the development of automatic metro operation.

## 1. Introduction

An automatic metro is a complex distributed socio-technical system [1,2], the traffic dispatching tasks in which often need to be carried out jointly by personnel from the operation control center (OCC), stations, and trains [3,4]. All participants compose a dynamic human–automation team with traffic tasks as the core [5]. Task complexity, which is a key indicator to describe task characteristic, has been deemed to determine team performance [6,7]. Especially in some emergency scenarios, the complexity of the traffic dispatching team task might directly affect the safety of the metro system [8]. Taking China as an example, many metro lines have adopted or will adopt the fully automatic operation (FAO) technology, such as the Beijing subway Yanfang line, No. 3 line, Daxing airport express of Beijing, Zhengzhou metro line 3, etc. The automation rate of the system becomes very high. Almost 90% of the work is conducted by the system, which seems to reduce the workload of operators, but this is not the case [9,10].

Under normal situations, operators hardly need to participate in the train operation when it is under a high level of automation, such as GOA3 and GOA4; however, in the case of an emergency, the tasks of operators are no longer easy [11,12]. Meanwhile, as the tasks of drivers are replaced by the dispatching team of the OCC, the complexity of team tasks, the frequency of communication, and the dynamic of emergency disposal are significantly increased. At the same time, the system risks become uncertain with the redistribution of responsibilities [13]. Compared to the individual task, a team task has more interaction patterns, more frequent concurrent behaviors, and richer organizational structures [14].

With the increasing automation degree of the metro system, there will be three main grades of automation in the same metro network: GOA2, GOA3 and GOA4. Improvements in the system have changed the complexity of many tasks. Especially in GOA4, the driver is no longer on the train, and the scene information is obtained totally by remote interfaces. Compared with the low level of automation, the task performance mode of the traffic dispatching team has changed a lot. In the case of emergencies, automation technology results in more confirming and operating procedures. A human team is the last barrier for the safe operation of the system [15]. The higher the level of automation, the higher the requirement for the efficiency of personnel intervention under emergencies [16]. Therefore, the complexity of a team task becomes a critical issue in a highly automated system. A metro system is a highly dynamic system, and the safety risks in the operation process change rapidly. Moreover, it is necessary to guarantee the operation efficiency during rush hour. Therefore, with the upgrading of automation, how to describe and cope with the traffic dispatching team task complexity (TDTTC) and how the TDTTC affects system safety will become increasingly prominent problems.

The construction of task complexity became a variable of interest in the mid-1960s [17], and a large number of studies have been carried out in different fields, such as computer [18], nuclear power [19], aerospace [20], etc. However, it is only in recent years that the complexity of team task has been mentioned frequently [21]. Although existing models, such as [22,23], have been widely accepted, some studies have posed new challenges to the assumption of the concept of task complexity with multiple participants [21]. Task no longer refers to isolated objects of analysis but is affected by system environment, organization, and other factors, so the complexity of team task becomes difficult to describe for specific systems. Under normal situations in the automatic metro system, the workload of a team task is very low until eventually intervention is no longer required. However, in abnormal and emergency situations, people swap with the role of the automation system. The communication between team members is more frequent, and the information transmission is more diversified, which increase task complexity. It is difficult to say whether the improvement of automation degree has a positive or negative impact on the task’s complexity [24].

The existing modeling approaches for task complexity, such as graph entropy [25] and the TC (task complexity) method [26], were mostly based on traditional descriptive or normative task analysis methods, such as hierarchical task analysis [27,28], which paid more attention to the decomposition of task structure and were effective in describing individual task complexity. For team task, therefore, cognitive work analysis (CWA) [29], which was oriented to the analysis of a socio-technical system, has better applicability. Since this approach is a formative method, it can be used to capture all possibilities of a system that lead to the complexity of a task, especially for some unpredictable scenarios [30]. The team cognitive work analysis method developed based on traditional CWA had proved its applicability in the multiplayer interaction analysis of a surgical team [31]. Meanwhile, in order to obtain the quantitative output of CWA for team, some studies began to combine CWA with nonlinear theory, and many new insights have been obtained [32].

As a formative method for work analysis, cognitive work analysis can support the analysis of complex socio-technical systems with the aim that in-depth understanding of the inter-relations of social systems and technical systems was required to fully appreciate how constraints act upon the working of system functions [33]. It is generally accepted that CWA can be divided into five main phases, each focusing on different constraint sets and presenting different perspectives on the system. Work domain analysis (WDA) can be used to model the possibilities for behavior given its constraints, including existing and unanticipated behavior, rather than describing actual behavior. Control task analysis (ConTA) combines the information processing of the actors as well as the task target of the man–machine system to model the tasks in the metro system so as to identify the cognitive and behavioral demand of the traffic dispatching team. Straight analysis (StrA) is used to study which potential strategies can be used to perform work activities under the constraints of automatic systems. Social organization and cooperation analysis (SOCA) can be used to divide tasks between system resources, including people and computers, and this dimension focuses on how teams communicate and collaborate. Worker competencies analysis (WCA) is used to analyse personnel capability requirements under system work requirements. WCA can help us determine whether the requirements obtained in the first four phases of the CWA exceed and match the capabilities of the dispatchers. At present, CWA is widely undertaken in the analysis of complex systems in the fields of nuclear power main control room [34], road safety [35], railway transportation [36], etc. 

The CWA approach clearly allows room for social and team interactions in the SOCA. However, the original CWA literature [37] left this particular level largely unspecified. Many studies tried to develop social organizational analyses for their work domains [38]. It became apparent that a single-phase model for SOCA may not be appropriate. Indeed, social organizational interactions have a complexity of their own and result in a broad set of social and functional constraints influencing not only WDA and ConTA but also StrA and WCA. For this reason, Ashoori et al. proposed that it can be useful to modify the traditional five-level CWA approach, whereby there is a parallel set of social or team models [39]. In team-CWA, it looks at past individual work to identify teamwork constraints in four levels: (a) team work domain analysis (team WDA), (2) team control task analysis (team ConTA), (3) team strategy analysis (team StrA), and (4) team worker competencies analysis (team WCA). The hypothesis proposed by Ashoori et al. to revise the CWA provided useful theoretical support for the technical route of combining qualitative analysis with quantitative analysis proposed in this paper.

There are a lot of parallel and nonlinear processes in dispatching team tasks under emergencies. With the traditional linear method, it is difficult to describe this kind of problem. The network theory method is used to solve the nonlinear problem. In a complex socio-technical system, team tasks cannot be well-analyzed by an event sequence-based approach because of the lack of analytical functionality to capture complex interactions in social technology systems [40]. In fact, the network model of team task is constructed using network theory, based on which the three-dimension conceptual model of team task complexity (TTC) is established [41]. Through the team task network model and three-dimension conceptual model of TTC, the source of the complexity of the team task response to the emergency scene can be clearly understood, and the task can be transformed into a network with event nodes and information clues as the edges. Therefore, the intrinsic properties of nonlinear systems can be described by the indicators of the network itself. The network theory abstracts the object into a system with many nodes and edges, and the intrinsic features of nonlinear systems can be described by the indicators of the network itself [42,43].

Aiming at the problem of team task complexity in the automatic metro system, this paper investigated the TDTTC through two stages by the methods of team-CWA and network characteristic analysis. The emergency situation was understood from the perspective of a socio-technical system, and the method framework from qualitative analysis to quantitative analysis was established. The method provided means for modeling and analyzing task characteristics of complex automated systems.

## 2. Method

According to the complexity dimension mentioned by Vicente [44], the following list of inter-related characteristics is intended to be broad enough to subsume the different types of complexity that we can find in automatic metro system: (a) large problem spaces, (b) social spaces, (c) heterogeneous perspectives, (d) distributed spaces, (e) dynamic spaces, (f) potentially high hazards, (g) many coupled subsystems, (h) automated systems, (i) uncertain data, (j) mediated interaction via computers, (k) disturbances. 

**Figure 1:**
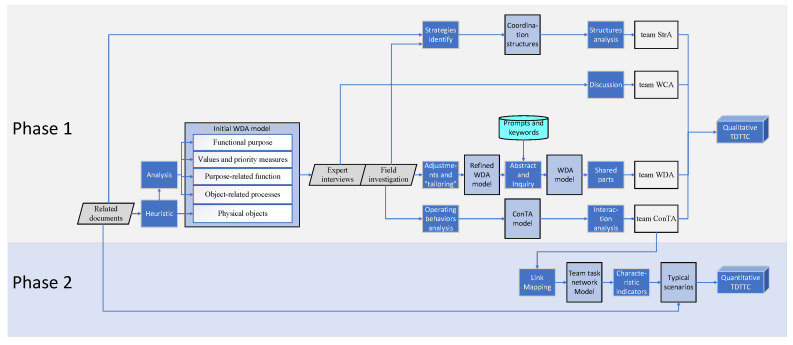
The build process of the TDTTC method.

The method consists of two phases. Phase 1 is qualitative TDTTC analysis, which is described by signal fault scenario. The second phase is quantitative TDTTC analysis. The method of this phase is to further model on the basis of team ConTA results in the first phase. However, in order to better compare the impact of automation on complexity, the screen door fault scenario is adopted for description in this phase. The entire build process is shown in Figure 1.

Different graphs are used in the method to represent different concrete processes. The grey diamond represents the data collection method, the dark blue rectangle represents the processing step, the light blue rectangle represents the intermediate part of the model, the white rectangle represents the four levels of team-CWA, and the blue cylinder represents the prompts and keywords used during the abstraction-level modeling. Details are provided below.

### 2.1. Data Collection Methods

The methods of field expert interview, field investigation, and document analysis were used to collect data. Data collection in this paper is carried out from three aspects:

(1) Related documents collection and review

From January to February 2021, we read the existing Beijing subway dispatching rules, Shanghai metro dispatching rules, operation manual of central dispatching terminal of automatic train monitoring system, RA11018 iTS 5002 Local MMI Operation Manual Guide, and RA11018 iTS 5001 Central MMI Operation Manual Guide. There are three uses for related documents. First, it is used to obtain physical object constraints of the initial WDA model, as shown in Figure 1. The more abstract the function of the model, the more likely it is to be inspired by stakeholders or by the literature on the purpose or objectives of the model. An understanding of the responsibilities of the model is often obtained through the relevant technical documentation. Then, the analysis of team SrA is also obtained by describing the responsibilities of different positions in specific tasks in the documents. Finally, the task flows of the screen door fault scenario under GOA1–GOA4 are described through the documents.

(2) Expert interview

From September to October 2020, we conducted 3 rounds of interviews with 5 experts in the field of rail transit operation. Two dispatchers came from the Beijing subway Yanfang line and 3 system designers came from Beijing Traffic Control Technology Company, of which 1 was a traffic dispatcher (5 years metro industry experience), 1 was a vehicle dispatcher (5 years metro industry experience), 1 was a system designer (3 years metro industry experience), 1 was a design deputy supervisor (6 years metro industry experience), and 1 was a design supervisor (11 years metro industry experience). The 3-round interviews were conducted in State Key Laboratory of Rail Traffic Control and Safety in Beijing Jiaotong University. All 5 experts participated in every round of the interview to make sure that we obtained more insights from each expert at each time. Expert interviews are used to make improvements to WDA and to obtain detailed information about the WCA team.

(3) Field investigation

In December 2020, we conducted a field investigation in the Beijing subway Yanfang line OCC three times. In order to master the operation of integrated monitoring system in OCC, we collected 1534 min of video materials of all functional operation interfaces and typical scenarios and conducted behavior analysis by video editing software. Field investigations also have three purposes. First, they are used for the WDA model to carry on the adjustment and tailoring; second, the strategies identify team StrA; third, the operating behaviors analysis data of team ConTA were obtained by field investigations.

### 2.2. Processing Steps and Intermediate Parts

(1) Heuristic

A general idea of the nature of the system constraints being analysed, which often involves creative thinking.

(2) Analysis

Determine the boundaries of the model. The scope of the boundary should include the system in as much detail as possible, but the analysis should still be manageable. The purpose of the analysis, the engineering constraints, and all the natural boundaries of the system need to be considered. Appropriate names will reflect natural boundaries. It has to be an event-independent perspective. In measuring what should be included in the WDA, the analyst can consider what the system is used for and what it does on it from the perspective of the actor.

(3) Adjustment and tailoring

Think critically and ignore the distractions of experience and background.

(4) Operating behaviors analysis

Conduct continuous action analysis of dispatcher’s operation through professional behavior analysis software. Observer XT 9.0 behavior observation software was adopted in this paper.

(5) Strategies identification

There are four types of team-related strategies [39]: structural strategy, operational strategy, coordination strategy, and team development strategy. Among them, the structured strategy inherits and builds on the possibility of behavior in the workspace and provides alternatives; operational strategy explores various optional behaviours to complete tasks; coordination structure identifies how different team structures are used to accomplish tasks; the team development strategy acknowledges that there are different possible options at different stages of the team experience of working together. Strategy identification selects and analyzes the above four strategies.

(6) Structures analysis

Analyze coordination structures of team StrA using the selected coordination strategy.

(7) Abstract and inquiry

The abstract dimension is determined by identifying the structural attribute of the behavior-subject environment. These structural attributes are divided into different types of concepts, and these concepts are organized into a means–end connection structure. The process of abstract and inquiry is obtained by the process of prompts and keywords.

(8) Shared parts

Analyze the parts of the WDA that members shared.

(9) Interaction analysis

To obtain the interactions between members during the task execution, which are divided into synchronous and asynchronous, see Section 3.3 for details.

(10) Discussion

Determine the contents of team WCA together with experts.

(11) Link mapping

The intermediate transformation link between qualitative and quantitative analysis, which is discussed in Section 5. 

### 2.3. Prompts and Keywords

The relationship between the abstract level and the prompts and keywords is shown in Table 1.

## 3. Team-CWA for TDTTC

### 3.1. Scenario Description

**Figure 2:**
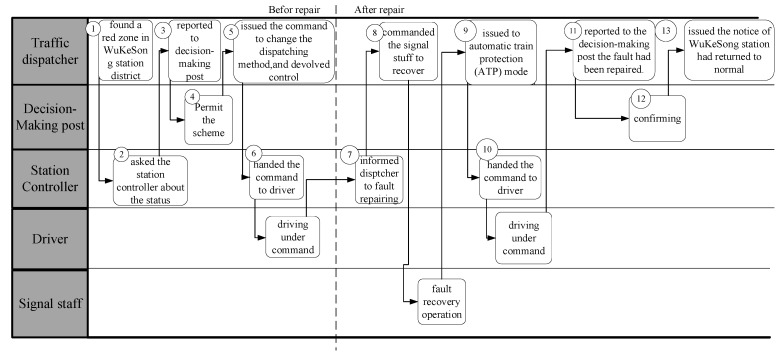
Workflow of team members in situation of signal failure.

A dispatcher in OCC found a red zone (i.e., the zone that the train cannot enter because of signal control system) in WuKeSong station district on the big screen at 14:00, so he immediately asked the station controller about the status of signal machine (i.e., signal) in WuKeSong station. After the confirmation, the dispatcher reported the situation to the decision-making post and decided to change the train running in the up and down districts of YuQuan Road station and WanShou Road station to the telephone blocking method and devolved control to the stations in the district. Subsequently, the dispatcher first confirmed the location of the up and down trains, checked the train number with the YuQuan Road station, told the controller of YuQuan Road station to change the order of telephone blocking from no. 2133, and told the driver to change the method of telephone blocking. Then, the dispatcher checked the train number with the WanShou Road station and told the WanShou Road station controller to hand in the blocking order from no. 1120 as well as told the driver to change the blocking driving method from no. 1120. The dispatcher of OCC then confirmed the location of the train in the fault district and told the train no. 1119 and 2132 where to stop; meanwhile, the dispatcher handed over the order of changing the telephone blocking method by WuKeSong station. Then, the dispatcher informed the online trains and all stations in the up and down sections districts from YuQuan Road to WanShou Road to change the telephone blocking method. The WuKeSong station controller, 14:25, informed the dispatcher that the fault recovery operation had been conducted. The dispatcher held all the trains from YuQuan Road to WanShou Road in the station and told signaling staff to recovery failure. After the failure recover, the dispatcher gave an order to change the train running in the up and down districts of YuQuan Road station and WanShou Road station to the telephone blocking method. The WuKeSong station controller was told to order train no. 2136 and no. 1123 to return to automatic train protection (ATP) mode. After that, the dispatcher reported to the decision-making post that the fault had been repaired. After the confirmation of the decision-making post, the dispatcher issued the notice that WuKeSong station had returned to normal to the online trains and all stations. Finally, the dispatcher in OCC issued an order to the stations between YuQuan Road and WanShou Road to take back control. The workflow of team members in the whole process is shown in Figure 2.

### 3.2. Team Work Domain Analysis

**Figure 3:**
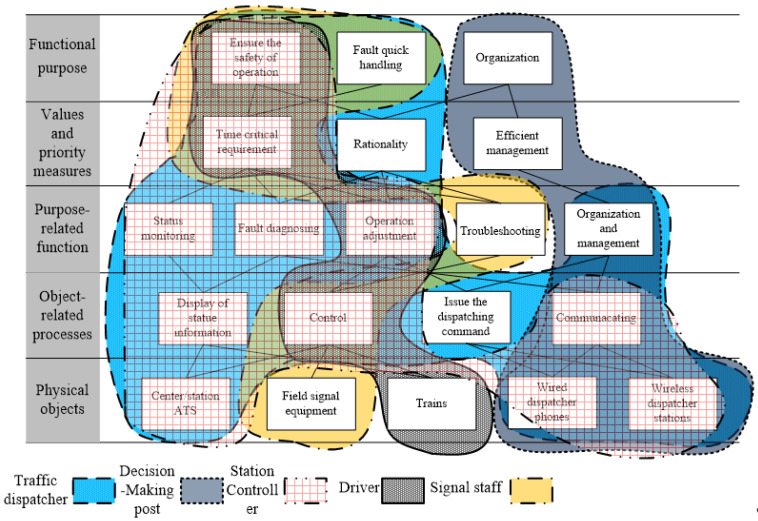
Abstract-level model for the traffic dispatching team.

Firstly, the method of abstraction level is adopted to establish the team work domain model of the traffic scheduling team, as shown in Figure 3. Members of the team include the traffic dispatcher in OCC, decision-making post, station controller, signal staff, and the drivers who are affected by the fault. Different types of members are distinguished by different colors.

The purposes shared by the traffic dispatching team are obtained through the functional purpose level. Ensuring traffic safety is the functional purpose of traffic dispatcher, station controller, driver, and signal personnel. Quick handling of faults requires the traffic dispatcher to make a correct solution for fault characteristics immediately, and the signal staff need to conduct quick maintenance to eliminate the signal fault after the fault occurs; the two roles share the purpose of fault handling. The decision-making post is solely responsible for the organization in the fault handling scenarios. 

The shared values, priorities, and principles are obtained by the values and priority measures level. Time critical requirement is a shared value of the traffic dispatcher, station controller, driver, and signal staff; rationality is a separate priority for the traffic dispatcher handling failures; efficient management is the principle to be followed by the decision-making post alone.

The shared functions are acquired through a purpose-related function level. Among them, a traffic dispatcher and a station controller jointly respond for the function of status monitoring and fault diagnosing. The operation adjustment function is shared by a traffic dispatcher, station controller, driver, and signal staff. The organization and management function is undertaken by the decision-making post alone; the signal staff is responsible for the troubleshooting function.

Team WDA boundary objects are obtained through the object-related processes level and physical objects level, which are center/station automatic train supervision (ATS), field signal equipment, trains, wired dispatcher phones, and wireless dispatcher stations.

### 3.3. Team Control Task Analysis

Traffic dispatching team is a distributed architecture which consists of two subteams: an OCC dispatcher team and a driving team. The OCC dispatcher team includes the decision-making post, dispatcher A, and dispatcher B; the members of the driving team are station controller of Wanshou Road, station controller of Yuquan Road, signal staff, and drivers of train no. 2133, 1120, 1119, 2132, 2136, and 1123. An interaction analysis list is obtained, which is shown as Table 2. The 13 events in the table are consistent with the numbers in Figure 2.

### 3.4. Team Strategies Analysis

Operation adjusting means are unique and fixed according to the design principle of a rail transit signal system, which is used to issue the dispatching command and control the trains and stations by ATS, dispatching telephones, and dispatching stations; therefore, there is no need to consider the structural strategies. Additionally, the given scenario is a signal fault scenario, which belongs to the range of abnormal scenarios, and the processing process in this case is a standard procedure of dispatching command operation; thus, the change of operational strategy does not need to be considered. Then, the effects of team development strategies do not need to be considered either because all the members of the team are qualified after long-term professional training. 

**Figure 4:**
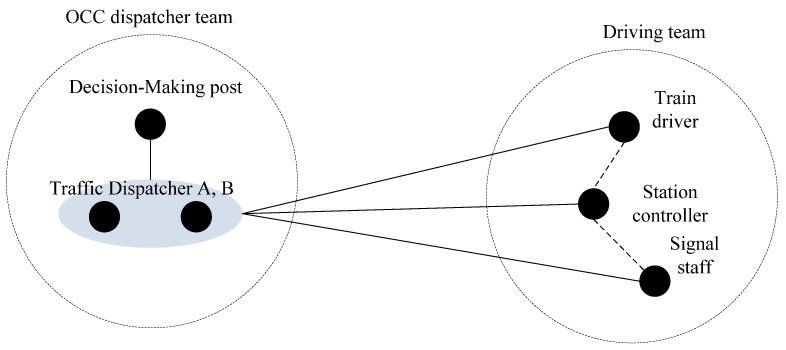
The coordination structures model for the traffic dispatching team task.

The coordination structures are analyzed in this case. In the signal fault handing scenario, the dispatcher is a core of the team who is responsible for fault diagnosing and conforming, command issuing, resource coordination and allocation, etc. Each step of troubleshooting has a dispatcher of participation, decision, and organizing, which forms the autocratic structure. In the OCC, the relationship between the dispatcher and the decision-making post is a hierarchical structure. In the driving team, the anarchistic structures caused by team task are formed in the relationship between the station controller and the driver as well as between the station controller and the signal staff. The whole traffic dispatching team is a composite structure, which can be seen in Figure 4.

It is easy to find that through this coordination structures model, in the whole team, dispatcher A and B bear almost all the control tasks, so the post is the core of the whole team. In the design of TDTTC, more attention should be paid to the effects of the traffic dispatcher on the entire team. The worker competencies of the dispatcher especially have a great influence on the completion of the task, which is obtained by team worker competencies analysis, as shown in the following section.

### 3.5. Teamworker Competencies Analysis

The team worker competencies analysis of the traffic dispatching team is obtained by the skill–rule–knowledge (SRK) inventory [45]. The functional and the social skills are also identified based on the SRK inventory. The results of the team worker competencies analysis are shown in the Table 3.

## 4. Quantitative Analysis of TDTTC for Emergency Situation

### 4.1. Network Model Construction for Team Task under Emergency

**Figure 5:**
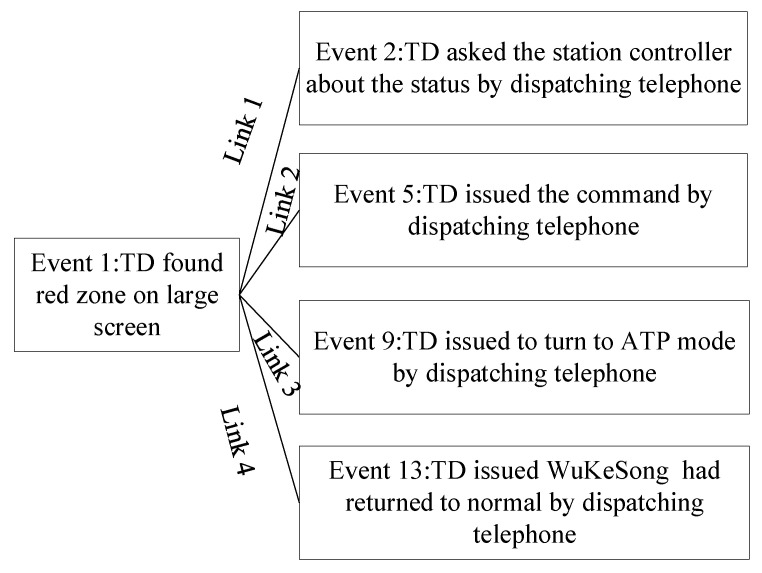
The mapping rules for the traffic dispatching team task.

According to the hypothesis of Hærem and Pentland [21] and the above analysis, this paper constructed the interactive results. Each event is linked to other events by information cues, as shown in Figure 5. Each event is an action performed by some actors at a specific time. The information clues generated by the event are processed by other actors. Take the links formed by event 1 as an example; event 1 is associated with event 2, 5, 9, and 13 through the information clue of the red zone being found. 

**Figure 6:**
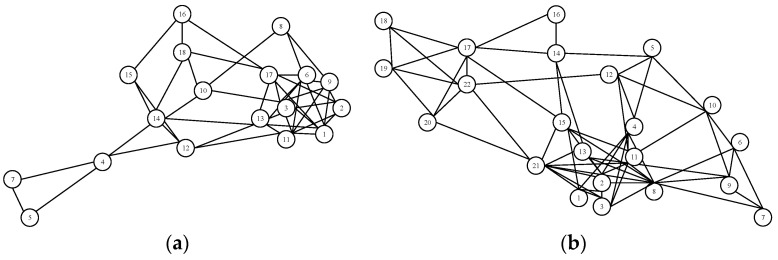

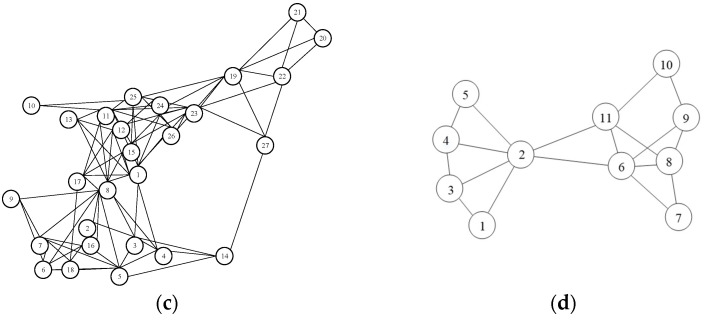
Team task complexity network models of screen door fault failures at GOA1–GOA4. (**a**) GOA1. (**b**) GOA2. (**c**) GOA3. (**d**) GOA4.

Using the mapping rules shown in Figure 5, the interactive relationships of the signal failure scenario can be established. Meanwhile, the traffic dispatching team task can be regarded as a complex network composed of node events; each node event contains a behavior agent and all the operations of the behavior agent in the event [46,47]. For example, event 2 “TD asked the station controller about the status by dispatching telephone” occurs in event 1 “TD found red zone on after a large screen”; event 2 can occur only after event 1 is completed. The two events are linked by the information clue of the timing of the red zone. When all the events have been traversed, the task of the entire traffic scheduling team is completed. Figure 6 shows the team task network models of screen door fault failures at GOA1–GOA4 by the mapping rules of Figure 5. 

When events are regarded as network nodes and information clues related to events are regarded as edges, each complex network model of traffic scheduling team tasks can be abstracted as an undirected network (*G*) whose basic elements include node set (*V*) and edge set (*E*).

$$G=\left\{V,E\right\}$$

$$V=\left\{v_{1},v_{2},\cdots v_{n}\right\},\;E=\left\{<v_{i},v_{j}>|v_{i},v_{j}\in V\right\}$$

### 4.2. Selection of Network Characteristic Index

Based on literature [48], the TDTTC is constructed into three dimensions, including nine types, which are, respectively, the irregularity types of team tasks; the information processing requirements type of team tasks and the target types of team tasks, which belong to the complexity dimension of problem space; the path entropy type, interaction type, information fuzziness type of team task belong to the complexity dimension of information connection; the scale type of team task, the variability type of team task and the characteristic function type of team task form the complexity dimension of structural characteristics. The relationship between TDTTC types and network characteristic index is further analyzed in this paper.

The types of complexity in the three dimensions are closely related to the network characteristics. The main factors that affect the complexity of the network are some aspects of the characteristics of nodes and edges, which are the basis of the realization of the task in the complexity of the team task. Therefore, the corresponding relationship between the network characteristic index and the complexity type of the team task can be established to explain the complexity of the team task network from the theoretical level.

Node degree and average degree are used to describe the scale of team task complexity network and the impact of some key nodes on the whole network. This index can be used to find the differences in the scale of different emergency situations as well as the differences in the path of information flow and processing needs. The related types include scale, variability, characteristic function, path entropy, and information processing requirements.

The agglomeration coefficient reflects the concentration effect brought by some nodes in the complex network of team tasks. In the actual process, these nodes are the key hubs of information, which are crucial for the security and reliability of the system. A high agglomeration coefficient indicates close correlation between events and high network operation efficiency; otherwise, it indicates low operation efficiency. However, it should be noted that in the process of an emergency situation, once these nodes are blocked or have poor circulation, related events cannot be completed, which may lead to accident risks. The related types include variability, information fuzziness, and information processing requirements.

The average shortest path length reflects the efficiency of information transmission between events of a team task. The shorter the shortest path, the better the correlation between events and the higher the efficiency of emergency scenario processing. Processing efficiency is very important for a dynamic subway system. In order to restore normal operation as soon as possible, it is necessary to consider the average shortest path length in emergency flow design. The related types include path entropy, interactivity, and irregularity.

The overall efficiency of the network is analyzed through the damage resistance. Damage resistance analysis is the ability to check whether the emergency situation can be coped with after random or deliberate removal of certain event nodes. The analysis result reflects the robustness of the task flow design. If the overall network efficiency decreases significantly after some nodes are removed, it indicates that these nodes are crucial for the completion of the emergency flow processing. Therefore, redundancy and backup should be considered at the beginning of the design. The related types are path entropy, interactivity, information ambiguity, and target. The calculation methods of each index are summarized in Table 4

### 4.3. Verification of Quantitative Analysis

In order to verify the feasibility of quantitative analysis, the emergency situation of screen door fault under different automation levels is selected. The screen door fault handling process is: when the screen door fault occurs, the driver (GOA1 and GOA2) or vehicle dispatcher (GOA3 and GOA4) must report to the traffic dispatcher immediately. The traffic dispatcher then informs station staff to do a screen door combined operation; meanwhile, the driver (GOA1 and GOA2) or passenger dispatcher (GOA3 and GOA4) broadcasts to passengers. Station staff report to OCC after troubleshooting and the traffic dispatcher issues an order to resume normal operations. The specific process varies according to the level of automation. Adopt the same process mentioned before to construct the complexity network of the screen door fault emergency task under different automation levels, as Figure 6 shows.

**Figure 7:**
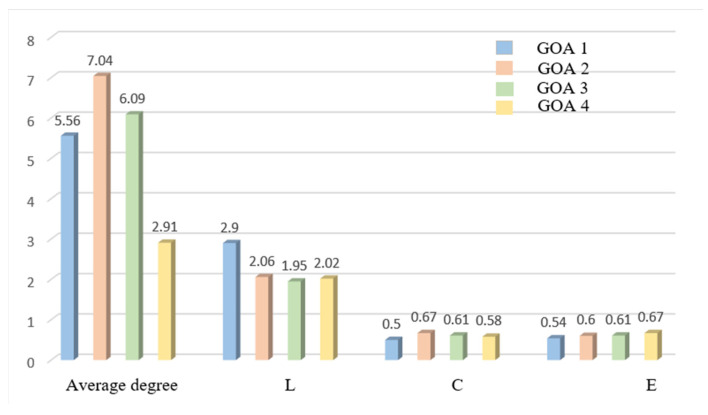
The statistical results of characteristics for shield door fault failures at GOA1–GOA4.

The results of the network characteristics of four automation levels of the screen door failure emergency procedures team task complexity indicators are shown in Figure 7.

It can be seen from the statistical results that GOA4 has the lowest node degree and average degree, while GOA3 has the highest node degree, which indicates that GOA3 has the closest connection in the emergency process of the failure of the flat screen door, while GOA4 has a looser connection between events. The values of node degree and average degree under GOA1 and GOA2 are both higher than GOA4. From the perspective of average shortest path length and aggregation coefficient, the average shortest path length under GOA4 is 2.9, which is the longest, and the minimum aggregation coefficient is 0.5. The agglomeration coefficient of GOA3 is as high as 0.67. The average shortest path length under GOA2 is the smallest, which is 1.94. As can be seen from the overall efficiency of the network, the team task complexity of the GOA4 shielded door emergency process under four automation levels is the lowest, and the network structure is relatively loose.

It is the role of the train driver in emergency procedure that leads to the contradiction between complexity and robustness, which is particularly true in the same situation under different levels of automation. Due to the change of driver involvement, the complexity of GOA4 < the complexity of GOA1 < the complexity of GOA2 < the complexity of GOA3, which is consistent with the simulation results that show that GOA4 owns the highest safety risks due to organizational factors. It is shown that if the complexity of system design needs to be reduced while system safety is improved after canceling the driver, the role of the driver and task migration must be rethought [49].

## 5. Discussion

TDTTC is affected by the results of Team-CWA from four dimensions. (1) The change of TDTTC may occur at different stages of the team task, namely, in the stage of signal fault detecting and diagnosing, there are problems of inconsistent information between the OCC and the stations in the failure districts, etc.; in the operating adjusting stage, the problems may be the complexity of lines and the communication under time pressure, etc.; in the fault recovery stage, the problems of high safety requirements, repeated confirmation, etc., may occur. All these problems may affect TDTTC. (2) The interaction complexity of the traffic dispatching team leads to changes of TDTTC. The stage and type of interaction can be understood through the decision wheel. The interaction complexity is affected by two aspects. On the one hand, it is the complexity of subtask execution; on the other hand, it is the complexity of interaction structure. The latter aspect provides a new idea to combine the Team-CWA with network theory. Some researchers have proposed that an important source of the complexity of a multiplayer task is the task network structure. (3) The coordination structures affecting TDTTC are obtained from team strategies analysis, which are variable structures according to different scenarios. The team strategy is different in each task scenario due to the different participation of team members in each task scenario. Whether this strategy is reasonable is an issue to be considered in affecting TDTTC. (4) The team worker competencies analysis defines the requirements of different levels for the traffic dispatching team. The complexity of the TDTTC subtask unit is undoubtedly influenced by the capacity of the actors. As a result, team worker competencies analysis has been able to identify the competency criteria for team members in different posts to discover the needs of the staff that influenced TDTTC.

Although qualitative analysis of TDTTC is obtained through team-CWA and insights from different dimensions can be investigated, multidimensional analysis brings some difficulties to the quantification of team tasks, that is, team task networking. Firstly, the interaction objects are realized through the behaviors of different members, who have different statuses in the task, which makes it difficult to standardize the measurement of task units, and the resulting network nodes have unequal status in the structure. Secondly, the interacting link is regarded as behavior, which makes it difficult for the interaction to form network structure, and thus, it cannot be further quantified. Thirdly, team task network needs to consider the theory of team task complexity. In some studies in the literature, the hypothesis of network construction based on events and information clues is put forward, which conforms to the concept assumption of task complexity by Wood [22]. The decision-making wheel model obtained by team-CWA is difficult to be directly applied to TDTTC.

Qualitative and quantitative results of TDTTC can be obtained through two-phase analysis, and the two phases are connected through the team task network modeling. This process is realized through decomposing the interactions obtained from the decision wheel to events and information clues. By comparing the results of task network characteristics analysis under different levels of automation, the feasibility of CWA to quantitative analysis was verified, which was proposed by many studies that showed that the results of human factors engineering analysis should be combined with the quantitative analysis path [50,51,52]. At the same time, this paper is an attempt to extend the application of a formative method. Although the formative method is not suitable for the modeling of specific tasks theoretically [53], the application scope of the CWA method is extended by combining event-independent and time-independent work analysis with task analysis dimension through the above mapping process. This builds a bridge from qualitative analysis to quantitative description of TDTTC, which provides the direction for further TDTTC quantification.

## 6. Conclusions

This paper proposes an analytical method of TDTTC from the perspective of a socio-technical system based on team-CWA and network feature analysis. The method is applied to study the changes of TDTTC under different levels of automation, which verifies the usefulness and correctness of the method. This method combines the advantages of the formative analysis method and the descriptive and normative analysis method and provides a new idea for the quantitative development of the human factors engineering method.

## Figures and Tables

**Table 1:** Prompts and keywords related to abstract level.

Abstract Level	Prompts	Keywords
Functional purpose	Why does the system exist or is the existence of the metro system necessary? What are the basic applications or outputs of the metro system? What is the value of the metro system? What is the purpose of the metro system design? What are the constraints or standards imposed on the metro system by an external entity or society? What external constraints should the metro system satisfy?	Reason, purpose, goal, intention, plan, service, output, exposition, role, value, practice, norm, law, policy, standard, discipline, guidance, requirement, rule, limitation
Values and priority measures	What are the fundamental principles or values that the metro system must consider? What are the criteria that must be met to achieve the functionality of the metro system? What criteria can be used to evaluate whether the metro system achieves its functional objectives? What criteria can be used to compare, sort, and assign purpose-related functionality?	Criteria, measures, success, effect, efficacy, reliability, quality, quantity, economy, consistency, frequency, probability, time, risk, budget, schedule, performance, results, indicators, price, testing, assessment, resources, principles, theorems, conventions, procedures, guidelines, requirements, rules, constraints
Purpose-related function	What functions must be supported to achieve functional goals? What are the functions provided by the physical objects of the metro system? What are the functions provided by processes related to physical objects? What are the physical resources put into the metro system for? What functions do people perform in the metro system?	Functions, operations, processes, activities, roles, responsibilities, jobs, tasks, positions, posts, positions
Object-related processes	What are the functional processes, functional capabilities, or constraints associated with metro system physical objects? What are the functional processes, functional capabilities, or constraints that are necessary for the physical objects of the metro system to achieve their purpose-related functions? What can system-related physical objects support?	Processes, capabilities, limitations, functionality, characteristics, performance, applications
Physical objects	What are the physical objects associated with the metro system? What are the physical objects necessary to achieve the object-related processes of the metro system? What are the physical objects necessary to implement the object-related processes of the metro system? What are the physical objects that need to be represented in the metro system?	Equipment, tools, artifacts, premises, infrastructure, facilities, installations, personnel, geographical forms, assets, resources

**Table 2:** Interaction analysis list.

Event	Member	Control Task	Boundary Objects	Synchronous and Asynchronous	Distribution
1	TD A, WuKeSong SC	Status monitoring, Fault diagnosing	Center/station ATS, dispatcher phones, dispatcher stations	Synchronous	OCC dispatcher team, driving team
2	TD A, WuKeSong SC	Fault diagnosing	Center/station ATS, dispatcher phones, dispatcher stations	Asynchronous	OCC dispatcher team, driving team
3	TD A, DM	Organization and management, Operation adjustment	Dispatcher phones, dispatcher stations	Synchronous	OCC dispatcher team
4	DM, TD A	Organization and management, Operation adjustment	Dispatcher phones, dispatcher stations	Synchronous	OCC dispatcher team
5	TD B, WanShou Road SC, YuQuan Road SC, WuKeSong SC	Status monitoring, Operation adjustment	Center/station ATS, dispatcher phones, dispatcher stations	Synchronous	OCC dispatcher team, driving team
6	WanShou Road SC, YuQuan Road SC, no. 2133 D, no. 1120 D	Operation adjustment	Dispatcher phones, dispatcher stations	Asynchronous	OCC dispatcher team, train team
7	WuKeSong SC, TD A	Status monitoring, Operation adjustment	Center/station ATS, dispatcher phones, dispatcher stations	Synchronous	OCC dispatcher team, driving team
8	TD A, SS	Operation adjustment, Troubleshooting	Center/station ATS, dispatcher phones, dispatcher stations	Synchronous	OCC dispatcher team, driving team
9	TD A, WanShou Road SC, YuQuan Road SC, WuKeSong SC	Status monitoring, Operation adjustment	Center/station ATS, dispatcher phones, dispatcher stations	Synchronous	OCC dispatcher team, driving team
10	WuKeSong SC, no. 2136 D, no. 1123 D	Operation adjustment	Dispatcher phones, dispatcher stations	Asynchronous	driving team
11	TD A, DM	Organization and management, Operation adjustment	Dispatcher phones, dispatcher stations	Synchronous	OCC dispatcher team
12	DM, TD A	Organization and management, Operation adjustment	Dispatcher phones, dispatcher stations	Synchronous	OCC dispatcher team
13	TD A, WanShou Road SC, YuQuan Road SC, WuKeSong SC	Operation adjustment	Dispatcher phones, dispatcher stations	Synchronous	OCC dispatcher team, driving team

Abbreviations: TD, traffic dispatcher; DM, decision-making post; SC: station controller; D: driver; SS: signal staff.

**Table 3:** Team worker competencies analysis.

Team Members		Skill-Based Behavior	Rule-Based Behavior	Knowledge-Based Behavior	Social Skills Required	Functional Skills Required
OCC dispatcher team	DM	A reasonable judgment is formed quickly through combining the treatment scheme proposed by the dispatcher with the specific fault situation.	Evaluate and give feedback for the scheduling plan of the dispatcher at any time and give guidance and help when needed.	Monitoring the ATS information and process of dispatcher continuously when the dispatcher makes operation adjustments.	Professional, self-starting, and able to provide knowledge with fewer resources.	Experienced dispatcher or driver with extensive field experience.
	TD	Experienced dispatchers should be able to quickly judge the occurrence of abnormal conditions and quickly diagnose faults through the information displayed on ATS.	The dispatcher should consider reasonable countermeasures according to the actual fault situation and flexibly apply the scheduling strategy on the premise of ensuring safety. For example, after receiving the notification that signal recovery can be conducted, the dispatcher should take safe and reliable signal recovery operation.	The dispatcher should quickly respond to the fault situation, form the correct fault treatment scheme, confirm the stations and vehicles involved in the fault according to the treatment process, and issue the correct scheduling command.	Cooperative, perceptive, and good communication skills. Be firm and confident in the team role. Professional, self-starting, able to provide knowledge with fewer resources.	Rich scheduling experience, be able to handle emergencies, have good psychological quality.
driving team	SC	Experienced station control personnel should quickly identify and judge the abnormal information and quickly form the diagnosis of the fault cause.	The station controller should quickly grasp and understand the results of operation adjustment, actively cooperate with the signal personnel to deal with the fault, and pay attention to the real-time change of the scene in the process of fault treatment.	The station controller should promptly respond to the dispatcher’s confirmation, inquiry, and dispatch command after the fault occurs and hand over the running order to the train driver in a timely and accurate manner.	Cooperative, perceptive, and good communication skills. Be firm and confident in the team role.	Station personnel who have received professional training.
	D	The driver should quickly identify the abnormal information and report the fault district in a timely manner.	Pay attention to the actual situation in the fault district continually and make quick responses in emergencies.	Regulate driving according to the dispatch command and driver’s manual.	Cooperative, perceptive, and good communication skills. Be firm and confident in the team role.	Trained drivers.
	SS	After receiving the troubleshooting task, the cause of the fault can be determined quickly.	Make a reasonable response to the difficult faults.	Ensure the safety of the premise, quickly troubleshoot, and accurately report to the station control.	Cooperative, perceptive, and good communication skills. Be firm and confident in the team role.	Signal staff who have received professional training.

**Table 4:** Index and its calculation formula details table.

Index	Calculate
Node degree and average degree	$k_{ij}=\sum_{j}a_{ij}$, $a_{ij}$ is the number of connected edges between node *i* and node *j.*
The average shortest path length (*L*)	$L=\frac{1}{C_{n}^{2}}\sum_{1\leq i\leq j\leq N}d_{ij}$, *N* is the total number of nodes in the network, and $d_{ij}$ is the distance between two adjacent nodes.
The agglomeration coefficient (*C*)	$C_{i}=\frac{E_{i}}{k_{i}(k_{i}-1)}$, $k_{i}\geq 2$, where $E_{i}$ is the actual number of connected edges in adjacent nodes of *i* and $k_{i}$ is the number of adjacent nodes of node, and the network agglomeration coefficient is: $C=\frac{1}{n}\sum_{v_{i}\in V}C_{i}$
The overall efficiency of the network (*E*)	$E=\frac{1}{N(N-1)}\sum_{i\neq j}d_{ij}$, *N* is the total number of nodes in the network, and $d_{ij}$ is the distance between two adjacent nodes.

## Data Availability

The data presented in this study are available on request from the corresponding author.

## Abbreviations

TDTTC	traffic dispatching team task complexity
Team-CWA	team cognitive work analysis
GOA	grade of automation
OCC	operation control centre
FAO	fully automatic operation
TC	task complexity
CWA	cognitive work analysis
WDA	work domain analysis
ConTA	control task analysis
StrA	straight analysis
SOCA	social organization and cooperation analysis
TTC	team task complexity
WCA	worker competencies analysis
